# Soft porous silicone rubbers with ultra-low sound speeds in acoustic metamaterials

**DOI:** 10.1038/srep40106

**Published:** 2017-01-05

**Authors:** Abdoulaye Ba, Artem Kovalenko, Christophe Aristégui, Olivier Mondain-Monval, Thomas Brunet

**Affiliations:** 1 Univ. Bordeaux - CNRS - Bordeaux INP, I2M, Talence, 33405, France; 2 Univ. Bordeaux - CNRS, CRPP, Pessac, 33600, France

## Abstract

Soft porous silicone rubbers are demonstrated to exhibit extremely low sound speeds of tens of m/s for these dense materials, even for low porosities of the order of a few percent. Our ultrasonic experiments show a sudden drop of the longitudinal sound speed with the porosity, while the transverse sound speed remains constant. For such porous elastomeric materials, we propose simple analytical expressions for these two sound speeds, derived in the framework of Kuster and Toksöz, revealing an excellent agreement between the theoretical predictions and the experimental results for both longitudinal and shear waves. Acoustic attenuation measurements also complete the characterization of these soft porous materials.

Since the pioneering works of Liu *et al*. about locally resonant sonic materials^1^, now so-called acoustic metamaterials^2^, the soft silicone rubber materials have brought renewed attention due to their supposed ‘ultra-low’ longitudinal sound speed^3^, ^4^, ^5^, ^6^, ^7^. When shaped as spherical particles, these ‘ultra-slow’ materials may theoretically act as strong Mie-type resonators leading to double negativity^8^. Few years later, Still *et al*. showed that such extremely low “longitudinal sound speed *c*_*L*_ ≈ 23 m/s is beyond any physically meaningful value in polymer science”^9^. These authors reported a combination of experiments revealing that the longitudinal sound speed is about 1000 m/s as expected for these homogeneous materials that are dense and non-compressible. On the other hand, gas-filled porous materials may exhibit very low longitudinal sound speeds *c*_*L*_ since these porous media may be both soft (*i.e*. compressible) and dense (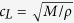, with *M* the longitudinal elastic modulus and *ρ* the mass density). For example, the longitudinal sound speed *c*_*L*_ in silica aerogels was measured at 100 m/s in ultrasonic experiments^10^. Following the original idea of Li and Chan based on the strong (monopolar and dipolar) Mie-type resonances of ‘ultra-slow’ resonators^4^, Brunet *et al*. have proposed to use porous polymer particles as key elements for a new class of locally resonant materials, so-called soft acoustic metamaterials^11^. The acoustic properties of these ‘ultra-slow’ particles have to be precisely known in order to give full account of exotic wave properties such as negative acoustic index^12^. For example, the thorough knowledge of the acoustic properties of silica aerogels at ultrasonic frequencies^13^ has recently enabled to interpret the emergence of two negative bands in soft three-dimensional acoustic metafluids made of porous silica particles^14^.

In this study, we report a complete ultrasonic characterization of soft porous silicone rubber materials for which no experimental data are up to now available. The elastic wave propagation in porous media is not a trivial issue and has been the subject of a large amount of works for many decades^15^. However, in the case where the incident wavelength *λ*_0_ of the propagating elastic wave is much longer than the pore size *a*, various more-or-less simple models have been reported to describe the effective properties of porous materials in the quasi-static limit^16^. In this context, we propose to use the model of Kuster and Toksöz^17^, in order to analyze the measured ultrasonic properties of soft porous silicone rubber materials with controlled porosities *ϕ* ranging from 0% to 35% (Fig. 1).

The sound-speed measurements reveal a sudden drop of the phase velocity *c*_*L*_ with the porosity *ϕ* for longitudinal waves, while the transverse phase velocity *c*_*T*_ remains constant as shown in Fig. 1. The strong dependence of *c*_*L*_ on *ϕ* has been attributed to the very low value of the shear modulus *G*_0_ (~1 MPa) of the soft elastomeric skeleton (with *ρ*_0_ ~ 1000 kg/m^3^), compared to that of its bulk modulus *K*_0_ (~1 GPa) as reported by Zimny *et al*.^18^. The interpretation of the porosity dependence of the longitudinal phase velocity was issued from the low-frequency approximation of a multiple scattering theory, referred to as Waterman-Truell model^19^. Here, we propose to analyze the porosity dependence of the phase velocities for both longitudinal and transverse waves with a simpler model based on a long-wavelength single scattering theory, referred to as Kuster-Toksöz model^17^. This model provides the effective elastic moduli and mass density (*K, G* and *ρ*) of the porous material as a function of its porosity *ϕ* in the quasi-static limit, *i.e*., when the pore size *a* is much smaller than the incident wavelength *λ*_0_. That is the case here since *a* ≤ 30 *μ*m and *λ*_0_ ≥ 1 mm for frequencies *f* lower than 100 kHz that are considered in this study.

Following the approach developed by Ament^20^, Kuster and Toksöz theoretically treated the propagation of seismic waves in two-phase media to determine the elastic (bulk and shear) moduli *K* and *G* of the composite medium given the properties, concentrations, and shapes of the inclusions and matrix material. In the long-wavelength limit, they obtained the composition laws for the effective mass density *ρ* and elastic moduli *K* and *G* (see equations 19, 20 and 21 in ref. 17). For air-filled porous *elastomeric* materials with moderate porosities (*ϕ* ≤ 50%), the mechanical properties of air cavities (*ρ*_1_, *G*_1_, *K*_1_) have no influence on the effective parameters of the porous material since 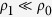, *G*_1_ = 0, 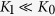 and can then be neglected. Since 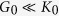 here, the relations established by Kuster and Toksöz can be simplified, providing thus the effective parameters for the porous material:


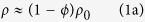



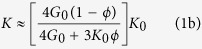



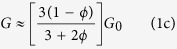


Therefore, the phase velocities for both longitudinal 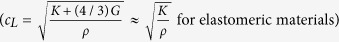 and transverse 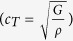 waves can then be easily deduced from the phase velocities of the elastomeric matrix 
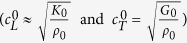
 as following:


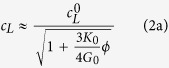



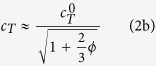


According to Eqs 2(a) and 2(b), *c*_*L*_ and *c*_*T*_ have been calculated as a function of the porosity *ϕ* by using the measured mechanical properties of our soft silicone rubber material (*ρ*_0_ = 1040 kg/m^3^, *K*_0_ = 1.2 GPa and *G*_0_ = 0.3 MPa). Figure 1 shows the comparison between these theoretical predictions (solid lines) and experimental data (open symbols), revealing an excellent agreement for longitudinal and transverse waves. Contrary to *c*_*T*_ which weakly depends on *ϕ*, as shown in Eq. 2(b), the sudden drop of *c*_*L*_ is due to the huge ratio 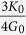 (=3000) as shown in Eq. 2(a). In these porous *elastomeric* materials, the longitudinal phase velocity is less than 100 m/s for a porosity of the order of a few percent, that has never been reported in other porous media. For example, silica aerogels require porosities higher than 90% to exhibit such low sound speeds^21^.

In Fig. 1, we also plotted the theoretical predictions issued from the long-wavelength approximation of the Waterman-Truell model as recently done^18^. It is worth noting that such a much more sophisticated model, based on multiple scattering theory, provides the same results as those obtained with the Kuster-Toksöz model based on single scattering theory. Thus, this simpler (Kuster-Toksöz) model is accurate enough to account for the strong porosity-dependence of the phase velocities of elastic (longitudinal and transverse) waves propagating in soft porous silicone rubber materials.

Attenuation coefficients *α* were also measured as a function of the frequency *f* and fitted with power laws (*α* = *α*_0_*f*^* n*^). For the longitudinal waves propagating in the *non*-*porous* sample (*ϕ* = 0%), we found *α*_*L*_ = 3.10^1^ *f*^ 1.4^ as shown in Fig. 2(a). The *n*-exponent value, of between 1 and 2, is characteristic of out-of-equilibrium media such as soft materials or soft tissues^22^. Furthermore, the attenuation coefficients *α*_*L*_ and *α*_*T*_ for both longitudinal and transverse waves were measured for all porous sample (see Table 1). As shown for *ϕ* = 35% in Fig. 2(b), the best fitting curves also provide exponents *n* of about 1.5, as observed for the non-porous sample, but the order of magnitude of *α*_0_ is a thousand times greater, that is probably due to scattering losses. The Kuster-Toksöz model only providing quasi-static expressions for the effective properties of the porous media, it is not expected to give full account of dynamical (frequency-dependent) quantities such as attenuation coefficients. However, the fitting parameters found here (*α*_*L*_ = 8.10^4^ *f*^ 1.4^ and *α*_*T*_ = 2.10^5^ *f* ^1.5^ for *ϕ* = 35%) are very close to previous ones measured in other soft porous silicone rubbers prepared using UV polymerization with an emulsion-templating procedure^12^,^18^.

In summary, we have reported a complete characterization of the acoustic properties of soft porous silicone rubber materials at ultrasonic frequencies (see Table 1). Measurements have revealed a drastic decrease of the longitudinal phase velocity with the porosity while the transverse phase velocity remains constant. The long-wavelength model of Kuster-Toksöz gives a fulfilling insight of such unusual behavior that has never been observed in any rigid porous media. The experimental data here reported, as well as the simple analytical expressions given for the sound speeds, may serve for various areas such as acoustic metamaterials^2^, for which soft particles made of ‘ultra-slow’ materials have proven to be interesting key acoustic resonators^23^.

## Methods

**Figure 1:**
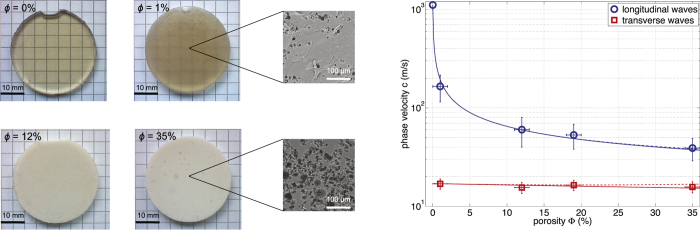
(left) Photographs of soft porous silicone rubbers with various porosities *ϕ* ranging from 0% to 35% and SEM micrographs of the porous structure for two samples with *ϕ* = 1% and 35%. The photograph of the 19%-sample is not shown here because it has the same appearance as the 12%-sample. (right) Phase velocities *c* versus porosity *ϕ* for both longitudinal 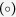 and transverse 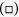 elastic waves. Solid lines refer to the Kuster-Toksöz model (Eqs 2(a) and 2(b)) with *K*_0_ = 1.2 GPa, *G*_0_ = 0.3 MPa, *ρ*_0_ = 1040 kg/m^3^ for the soft silicone rubber matrix, and *K*_1_ = 0.14 MPa, *G*_1_ = 0, *ρ*_1_ = 1 kg/m^3^ for the air cavities. Dash lines refer to the Waterman-Truell model (equations not shown here) with the same material input parameters.

The soft porous silicone rubbers used in this work were synthesized via an emulsion-templated procedure^18^,^24^. The silicone network was formed by a polyaddition reaction of SiH and SiVi in a mixture with a platinum-based catalyst. All products were obtained from BlueStar Silicones Co. Water-in-PDMS emulsions were prepared by mechanical stirring using a silicone-based Silube surfactant (Siltech). The emulsions were poured into 35 mm-diameter disk-like molds and stored at room-temperature until complete reaction (about four hours). Drying of samples led to soft porous silicone rubbers with pore sizes *a* ranging from 1 to 30 *μ*m, which correspond to the sizes of initial emulsion droplets. The porosity of the final materials was thus controlled by adjusting the volume of the dispersed water phase in the emulsions. The macroporous structure of all samples was confirmed by Scanning Electron Microscope pictures (TM-1000, Hitachi) as shown in Fig. 1. The mass density *ρ* for various porosities *ϕ* has been determined from the mass and the volume of the samples, and has been found to satisfy the mixture rule, Eq. 1a. Note that the mechanical properties of the *non*-*porous* silicone rubber were characterized using direct static mechanical measurements, providing *ρ*_0_ = 1040 kg/m^3^, *K*_0_ = 1.2 GPa and *G*_0_ = 0.3 MPa, for the mass density, the bulk and shear moduli, respectively. These values are consistent with those reported in the literature for rubber elastic polymer polydimethylsiloxane^25^,^26^.

The ultrasonic characterization of soft porous silicone rubber materials was performed for various porosities *ϕ* ranging from 0 to 35%. For a given *ϕ*, large monolithic samples (35 mm in diameter) were prepared with two different thicknesses *d* of a few millimeters. Each sample was then placed between two identical large broadband ultrasonic (US) transducers (emitter and receiver) with a diameter of 25 mm, *i.e*., smaller than the sample diameter. For longitudinal waves, we used two immersion transducers (Olympus V301), whereas two shear-wave transducers (Sonaxis CMP79) were used to generate and detect transverse waves. The US transducers were placed face to face and mounted on a linear manual stage, allowing the precise measurement of the sample thickness *d* with an uncertainty of about 10 *μ*m. Rather than using a coupling fluid between the US transducers and the sample that could penetrate its porous structure, each sample was first slightly pre-stressed by means of the transducers, and then relaxed before each measurement to ensure a good mechanical contact. The emitting transducer was excited with short (broadband) pulses generated by a pulser/receiver (Olympus, 5077PR) that was also used to amplify the electric signal recorded by the receiving transducer before its acquisition on a computer via a waveform digitizer card (AlazarTech, ATS460).

Figures 3 and 4 show typical experimental results, here obtained for both longitudinal and transverse waves propagating in a soft porous silicone rubber material with *ϕ* = 35%. In this case, the sample thicknesses *d* were 2.2 mm and 3.7 mm. The transmitted signals were first temporally filtered, keeping only the two first oscillations, to remove the electric pulses observed at short time and the multiple reverberations between the two US transducer surfaces (see echoes on Figs 3 and 4(a) and (b)). Such a cut-off protocol is appropriate since the signal waveform is not distorted significantly during the propagation within the material, as illustrated in Figs 3 and 4(c) and (d). Then, Fast Fourier Transforms (FFTs) were performed on these two gated signals to extract the corresponding phase and amplitude spectra (Figs 3 and 4(e) and (f)). Knowing the thickness difference between the two samples (*δd* = 1.5 mm), the phase velocity *c*_*L*_ and the attenuation coefficient *α*_*L*_ were deduced as a function of the frequency as shown in Figs 3 and 4(g) and (h). The phase velocities for both longitudinal and transverse waves are found to be constant (*c*_*L*_ ~ 40 m/s and *c*_*T*_ ~ 15 m/s) within the full width-half maximum bandwidth (see the grey area in Figs 3 and 4(g)). The dispersion effects are therefore negligible in this frequency range and then the use of the described cut-off procedure is justified here. At last, it is worth noting that the thermally-cured elastomer considered here exhibits phase velocities two times lower than that of previous soft porous silicone rubber materials polymerized by UV (*c*_*L*_ ~ 80 m/s and *c*_*T*_ ~ 40 m/s)^12^,^18^.

**Figure 2:**
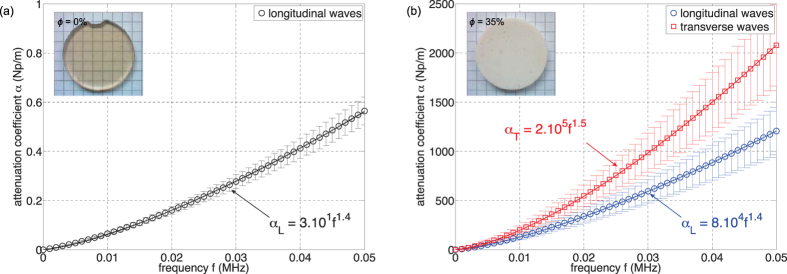
Attenuation coefficients versus frequency for both longitudinal 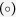 and transverse 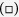 waves propagating through soft porous silicone rubber samples with *ϕ* = 0% (**a**) and *ϕ* = 35% (**b**). Solid lines refer to the best fitting curves obtained with power laws: *α* = *α*_0_* f*^* n*^, with *f* the frequency in MHz.

**Figure 3:**
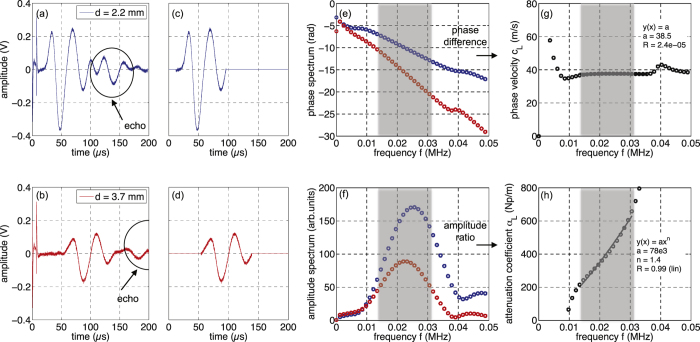
(**a** and **b**) Transmitted signals through soft porous silicone rubbers with *ϕ* = 35% for two different thicknesses: *d* = 2.2 mm and *d* = 3.7 mm for *longitudinal* waves. (**c** and **d**) Corresponding gated signals. (**e** and **f**) Phase and amplitude of the Fast Fourier Transforms of the two gated signals. (**g** and **h**) Phase velocity *c*_*L*_ and attenuation coefficient *α*_*L*_ vs frequency *f* deduced from FFTs; the solid lines correspond to the fits obtained from highlighted-grey experimental data.

**Figure 4:**
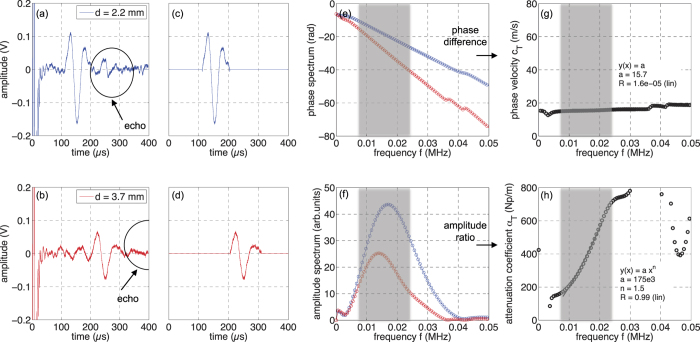
(**a** and **b**) Transmitted signals through soft porous silicone rubbers with *ϕ* = 35% for two different thicknesses: *d* = 2.2 mm and *d* = 3.7 mm for *transverse* waves. (**c** and **d**) Corresponding gated signals. (**e** and **f**) Phase and amplitude of the Fast Fourier Transforms of the two gated signals. (**g** and **h**) Phase velocity *c*_*T*_ and attenuation coefficient *α*_*T*_ vs frequency *f* deduced from FFTs; the solid lines correspond to the fits obtained from highlighted-grey experimental data.

These experiments were conducted ten times under the same experimental conditions to test the reproducibility and to get error bars on phase-velocity and attenuation measurements, shown for instance in Figs 1 and 2. The same experimental protocol was used to extract the phase velocities *c*_*L*_ and *c*_*T*_ for all other samples with various porosities. Note that it was not possible to measure the transverse phase velocity *c*_*T*_ for the non-porous material (*ϕ* = 0%) since the shear-waves are much more attenuated in these materials in comparison with the longitudinal waves. Despite the use of shear wave transducers, longitudinal modes are indeed still generated and completely dominate the shear modes, thus making their detection impossible.

## Additional Information

**How to cite this article**: Ba, A. *et al*. Soft porous silicone rubbers with ultra-low sound speeds in acoustic metamaterials. *Sci. Rep.* **7**, 40106; doi: 10.1038/srep40106 (2017).

**Publisher's note:** Springer Nature remains neutral with regard to jurisdictional claims in published maps and institutional affiliations.

## Figures and Tables

**Table 1:** Acoustic properties (phase velocities *c* and attenuation coefficients *α*) for soft porous silicone rubber materials with various porosities *ϕ* ranging from 0% to 35% for both longitudinal (_*L*_) and transverse (_*T*_) waves.

**ϕ (%)**	*c*_*L*_ (m/s)	*α*_*L*_ (Np/m)	*c*_*T*_ (m/s)	*α*_*T*_ (Np/m)
0	1110	30 *f*^ 1.4^	x	x
1	165	61.10^3^ *f*^ 1.4^	16.9	x
12	60	65.10^3^ *f*^ 1.5^	15.5	165.10^3^ *f*^ 1.4^
19	53	74.10^3^ *f*^ 1.6^	16.4	170.10^3^ *f *^ 1.6^
35	38.5	78.10^3^ *f *^ 1.4^	15.7	175.10^3^ *f*^ 1.5^

*f* is the frequency in MHz.
